# Transcriptional Response of *Mycobacterium tuberculosis* to Cigarette Smoke Condensate

**DOI:** 10.3389/fmicb.2021.744800

**Published:** 2021-10-15

**Authors:** Danicke Willemse, Chivonne Moodley, Smriti Mehra, Deepak Kaushal

**Affiliations:** ^1^Southwest National Primate Research Center, Texas Biomedical Research Institute, San Antonio, TX, United States; ^2^Tulane National Primate Research Center, Tulane University Health Sciences Center, Covington, LA, United States

**Keywords:** smoking, cigarette smoke, tuberculosis, *sigH*, mycobacterium

## Abstract

Smoking is known to be an added risk factor for tuberculosis (TB), with nearly a quarter of the TB cases attributed to cigarette smokers in the 22 countries with the highest TB burden. Many studies have indicated a link between risk of active TB and cigarette smoke. Smoking is also known to significantly decrease TB cure and treatment completion rate and increase mortality rates. Cigarette smoke contains thousands of volatile compounds including carcinogens, toxins, reactive solids, and oxidants in both particulate and gaseous phase. Yet, to date, limited studies have analyzed the impact of cigarette smoke components on *Mycobacterium tuberculosis* (*Mtb*), the causative agent of TB. Here we report the impact of cigarette smoke condensate (CSC) on survival, mutation frequency, and gene expression of *Mtb in vitro*. We show that exposure of virulent *Mtb* to cigarette smoke increases the mutation frequency of the pathogen and strongly induces the expression of the regulon controlled by SigH—a global transcriptional regulator of oxidative stress. SigH has previously been shown to be required for *Mtb* to respond to oxidative stress, survival, and granuloma formation *in vivo*. A high-SigH expression phenotype is known to be associated with greater virulence of *Mtb*. In patients with pulmonary TB who smoke, these changes may therefore play an important, yet unexplored, role in the treatment efficacy by potentially enhancing the virulence of tubercle bacilli.

## Introduction

Cigarette smoking is practiced by an estimated 20.7% of the world’s population, 80% of which belong to low- and middle-income countries. Smoking is considered an added risk factor for tuberculosis (TB), with nearly a quarter of the TB cases attributed to cigarette smokers in the 22 countries with the highest TB burden (WHO, 2019). Many studies have indicated a link between risk of active TB and cigarette smoke, with some predicting as much as a fivefold increase in the risk of active TB in smokers compared to non-smokers (Yu et al., 1988; Jha et al., 2008; Lin et al., 2009; Rao et al., 2012, 2017; Ozturk et al., 2014; Smith et al., 2015; Bronner Murrison et al., 2016). Smoking also significantly decreases TB cure and treatment completion and increases mortality rates (Chiang et al., 2012; Bonacci et al., 2013).

Whole cigarette smoke contains thousands of volatile compounds including carcinogens, toxins, reactive solids, and oxidants in both gaseous and particulate phase. Generation of whole cigarette smoke under laboratory conditions (containing both the soluble and insoluble fractions of cigarettes smoke that would be inhaled into the lungs of a smoker) is challenging. Two forms of cigarette smoke, cigarette smoke extract (CSE) and cigarette smoke condensate (CSC), are therefore routinely used for investigating the effect of cigarette smoke components *in vitro* and *in situ*. CSE is obtained by dissolving the water-soluble gas and particulate phase, generated from puffing a lit cigarette, in a phosphate-buffered saline solution. CSC consists of the water-insoluble fraction and is generated by passing the cigarette smoke through a filter pad and eluting the particles collected on the filter in a solvent. The compositions of CSE and CSC differ, with nicotine for instance making up 55.8% of CSC but only 8.98% of CSE (Mutepe et al., 2013). This is important to note when using either for investigating the effect of cigarette smoke. CSC has been used in many research studies to study the effect of cigarette smoke (Baboni et al., 2010; Mutepe et al., 2013; Cockeran et al., 2014; Semlali et al., 2014; Cholo et al., 2020).

Some of the compounds of cigarette smoke are pro- and others anti-inflammatory (Borgerding and Klus, 2005; Stampfli and Anderson, 2009; Kim et al., 2018). It increases the permeability of the epithelium, impairs mucociliary clearance, alters metabolism of cells, and induces oxidative and nitrosative damage to cell membranes and DNA—thereby ultimately causing apoptosis. Although smoking increases the production of pro-inflammatory cells, it compromises their ability to phagocytose and kill bacteria, thereby increasing bacterial burden (Stampfli and Anderson, 2009; Feng et al., 2011; Shaler et al., 2013; Agarwal et al., 2014; O’Leary et al., 2014; Aryanpur et al., 2016a,b; Bai et al., 2017). Smoking also causes genomic DNA mutations that are associated with cancers (Alexandrov et al., 2020).

The respiratory tract microbiota differs significantly between smokers and non-smokers (Charlson et al., 2010; Yu et al., 2017). Although many studies have investigated the effect of cigarette smoke on the host cells and responses, limited studies have been done to investigate the effect of cigarette smoke on human pathogens that commonly infect the respiratory tract. Cigarette smoke increases oxidative stress and biofilm formation in *Pseudomonas aeruginosa* (Chien et al., 2020), *Staphylococcus aureus* (Kulkarni et al., 2012; Hwang J. H. et al., 2016), *Streptococcus mutants* (Baboni et al., 2010), and the sino-nasal microbiota of smokers (Goldstein-Daruech et al., 2011). Increased cell adhesion, favoring persistent colonization, is observed in *S. aureus* and methicillin-resistant *S. aureus* (MRSA) (Kulkarni et al., 2012; Crotty Alexander et al., 2015; McEachern et al., 2015; Hwang J. H. et al., 2016). An upregulation of virulence genes and concomitant increased virulence is observed in *P. aeruginosa* and *S. aureus* (McEachern et al., 2015; Hwang J. H. et al., 2016; Kulkarni et al., 2016; Chien et al., 2020). Expression of virulence and adhesion factors are, however, not upregulated in *Streptococcus pneumoniae* (Manna et al., 2018), while biofilm formation is (Mutepe et al., 2013). *Mycobacterium tuberculosis* (Mtb) biofilm formation is also increased upon exposure to CSC (Cholo et al., 2020). Detoxifying enzymes, efflux pumps, and osmoregulator transporters, which can provide a direct defense against the cigarette smoke, are upregulated, while fatty and lipoteichoic acid synthesis is downregulated in *S. pneumoniae* (Manna et al., 2018). These different responses could suggest a bacterial specific cigarette smoke response.

It was suggested that the aforementioned changes may be heritable and therefore likely due to genetic modifications brought about by the cigarette smoke exposure. Resistance to antimicrobial agents is for instance observed as a result of cigarette smoke exposure (Miyahara et al., 2011; Crotty Alexander et al., 2015, 2018; Hwang J. H. et al., 2016; Hwang J. Y. et al., 2016; Chien et al., 2020). Although this resistance is linked to changes in the cell wall in MRSA (McEachern et al., 2015), cigarette smoke exposure was directly linked to clinically relevant mutations in drug [rifampicin (RIF) and ciprofloxacin] resistance genes of *P. aeruginosa* (Miyahara et al., 2011). In *Salmonella typhimurium*, the induction of mutant variants is also observed upon exposure to cigarette smoke components (Claxton et al., 1989; Aufderheide and Gressmann, 2007, 2008). This mutability is smoke concentration, composition, time of exposure, and strain dependent (Mizusaki et al., 1977; Morin et al., 1987; Camoirano et al., 1994; Yim and Hee, 2001; Yim et al., 2002; Aufderheide and Gressmann, 2007, 2008; Roemer et al., 2016; Thorne et al., 2016). It was previously suggested that cigarette smoke should also be considered as a mutagenic substance to *Mtb*, the causative agent of TB (McGrath et al., 2014). To our knowledge, the effect of CSC on the transcriptional response of Mtb has, however, not been investigated. In this study, the effects of CSC on the survival of *Mtb*, mutation frequency, and gene expression profile *in vitro* are explored.

## Materials and Methods

### Bacterial Culture Conditions

*Mtb* CDC1551 (BEI: NR-13649) was cultured in 7H9 containing 0.05% Tween-80 and supplemented with 0.2% glycerol and 10% oleic acid, albumin, dextrose, catalase (OADC) (Middlebrook) (subsequently referred to as 7H9 OADC). For exposure to CSC, 7H9 supplemented with 0.2% glycerol and dextrose (20 g/l) containing 0.05% Tween-80 (referred to as 7H9 dextrose) was used. CSC (Murty Pharmaceuticals, Lexington, KY, United States) was diluted fivefold in dimethyl sulfoxide (DMSO) to prepare working stock of 8 mg/ml which was added to 7H9 dextrose where indicated. Bacteria were also cultured on 7H11 agar (Difco) supplemented with 0.5% glycerol and 10% OADC (prepared in the laboratory: 0.06% oleic acid, 5% albumin, 2% dextrose, 0.004% catalase, 0.85% sodium chloride, and filter sterilized) (subsequently referred to as 7H11 agar) for colony-forming unit (CFU) enumeration. A RIF stock solution of 10 mg/ml was prepared in 10% DMSO 90% water solution and used where necessary at 2 μg/ml final concentration in 7H11 agar.

### MIC Determination

A serial twofold dilution series of CSC working solution (8 mg/ml in DMSO), DMSO, and 7H9 dextrose was prepared in 7H9 dextrose in a 96-well plate. *Mtb* cultures at day 4 of growth were diluted to optical density (OD) of 0.002 and equal volumes added to these serial dilutions (total 100 μl). After 3 weeks of growth at 37°C, resazurin (30 μl of a 0.02% stock) was added to each well and color development was allowed overnight. Pink wells indicated growth and blue, no growth. DMSO wells served as control for the effect of the CSC diluent on the growth of *Mtb* while the 7H9 dextrose wells served as positive *Mtb* growth controls. Three independent replicate experiments were done.

### Cigarette Smoke Condensate Exposure Survival Assays

Starter cultures were cultured in 7H9 OADC for 6 days, subcultured to OD 0.05, and allowed to grow for 4 days. Bacteria were collected, washed twice in 7H9 dextrose to remove catalase, resuspended in 7H9 dextrose, and sonicated to remove clumps. After filtering through a 0.2-micron cell strainer (Falcon) to remove clumps, the cultures were diluted to an OD of 0.2. For survival assays, these cultures were split into 10-ml cultures and exposed to 25, 50, or 75 μg/ml CSC or DMSO only (unexposed) for 3, 6, 24, and 48 h. Dilution series were prepared at each time point in phosphate-buffered saline (PBS) (pH 7.4) containing 0.5% Tween-80 and proper dilutions plated on 7H11 agar. CFUs were enumerated after 3–4 weeks. The percentage survival was calculated by dividing the CFU/mL of CSC exposed cultures with that of the DMSO-exposed (unexposed) cultures, which was set as 100% survival.

### *Mycobacterium tuberculosis* Mutation Frequency Determination

Rifampicin resistance is generally acquired by mutations in the *rpoB* gene of *Mtb* (Hirano et al., 1999). The acquisition of these mutations and subsequent ability of the bacteria to grow in the presence of RIF have been used in many studies to study the mutation frequency and rate in *Mtb* (McGrath et al., 2014). These assays were adapted for mutation frequency determination after exposure to CSC. Exposure to the diluent of CSC, DMSO, was taken as the mutation rate without exposure, to exclude any influence of the diluent. Preparation of cultures was done as for the CSC survival assays. Cultures were split into 30-ml cultures and exposed to 50 μg/ml CSC or equal volume of DMSO (unexposed) for 3 and 24 h. Dilution series of two separate cultures were prepared in PBS containing 0.5% Tween-80 and proper dilutions plated on 7H11 agar. Undiluted cultures (10 ml, 1 ml, and 100 μl) were also plated on 7H11 agar containing 2 μg/ml RIF. CFU enumeration was done after 4 weeks to exclude the presence of satellite colonies. The mutation frequency in the presence of CSC was calculated by using the average CFU in the two cultures exposed to CSC plated on 7H11 agar RIF (representing the RIF resistant mutants) and dividing it by the average CFU in the two cultures exposed to CSC plated on 7H11 agar (total bacterial count). The mutation frequency was calculated in the same way for the DMSO (unexposed) cultures. Three independent exposure experiments were done.

### RNA Extraction

Cultures (30 ml), prepared as for CSC mutation frequency analysis, were collected by centrifugation at 4,000 rpm, resuspended in 1 ml RNAProBlue solution (MP Biomedicals, Santa Ana, CA, United States), transferred to a 2-ml tube containing 0.2-mm matrix B beads (MP Biomedicals) and frozen at −80°C until use. After defrosting, bacteria were lysed by bead beating at 3,800 rpm for 3 cycles of 30 s each, using a BioSpec Mini-Beadbeater-24 and cellular debris removed by centrifugation at 15,000 × *g* for 15 min. Chloroform (300 μl) was added and the reaction vortexed for 10 s. The NucleoSpin RNA kit (Macherey-Nagel, Düren, Germany) was used for RNA extraction according to the manufacturer’s instructions. Even though an on-column rDNase treatment was done, the RNA was also treated with Turbo DNase from the TURBO DNA-free Kit (Ambion, Foster City, CA, United States) kit according to the manufacturer’s instructions. Following inactivation of the DNase using inactivation reagent (Ambion), the RNA Clean & Concentrator Kit (Zymo) was used to get rid of the DNase buffer and RNA was eluted in RNase-free water. Retreatment with Turbo DNase and cleanup using the RNA clean & concentrator kit were done, if DNA was still present. The concentration of RNA was determined using the Qubit RNA BR Assay Kit (Thermo Fisher Scientific, Waltham, MA, United States), and DNA contamination was assessed using the Qubit 1 × dsDNA HS Assay Kit (Thermo Fisher Scientific) on a Qubit Flex Fluorometer (Thermo Fisher Scientific). RNA quality was assessed using the 4200 TapeStation System by the Texas Biomedical Research Institute Molecular Core facility.

### RNA-Sequencing

The Zymo-Seq RiboFree Total RNA Library Kit was used for library preparation according to the manufacturer’s instructions. RNA (500 ng) was used and ribosomal depletion done for 30 min, followed by 14 cycles of PCR. RNA-seq was done using the Illumina NextSeq 500 V2.5 kit according to the manufacturer’s instructions on the NextSeq 500 machine by the UTHSCSA Genome Sequencing Facility. Sequencing reads were subjected to secondary data analysis using the Partek Flow (Partek Inc., Chesterfield, MO, United States) pipeline. In short, reads were filtered based on quality of Phred 30 with minimum read lengths of 25 nucleotides and aligned to the *Mtb* CDC1551 reference genome sequence (European Nucleotide Archive ASM858v1) using the RNA-seq aligner STAR v2.5.3a. Post-alignment quality assessment, transcript abundance estimation according to ASM858v1 transcriptome annotation by expectation-maximization algorithm, and sample read count normalization using Transcripts Per Kilobase Million (TPM) were done.

### Differential Gene Expression Analysis

Read count data (not normalized) were uploaded into the iDEP91 online RNA-seq analysis software (Ge et al., 2018). The minimal counts per million (CPM) were kept at default of 0.5 in at least one library. Count data were transformed for clustering and principal component analysis using EdgeR: log2 (CPM + c) and pseudocount (c) of 4. Gene IDs were not converted to Ensembl. Where multiple transcripts were present, full stops were replaced with underscores in the read count files to enable recognition of the transcripts as distinct. Missing-value imputation was done using the gene median. Principal component analysis was done. Differentially expressed genes were identified using the DESeq2 option on the iDEP91 software, with an FDR of 0.1 and minimum fold change of 2.0. Time was considered as a factor. Exposure to CSC was considered the main factor, reference DMSO used when comparing CSC vs. DMSO, and batch effect considered. The function of genes differentially expressed in the presence of CSC was determined by literature search on Google Scholar and the Mycobrowser website^1^. Raw and analyzed data can be accessed from GEO *via* the accession number GSE172041.

**TABLE 1:** Gene-specific primers used for RT-qPCR analysis.

Gene primer	Sequence (5′–3′)
SigAFor	TGCAGTCGGTGCTGGACAC
SigARev	CGCGCAGGACCTGTGAGCGG
MT3320For	GTCCAACGCCGAGCATTCCT
MT3320Rev	CCTGCAGCGCCTCTTTGATCT
MT1608For	AGGAGGAGATCGGTGCAGGT
MT1608Rev	ACCGAGGACCCGCAAATCAC
MT1259For	GCTACACCGCATCACCACCA
MT1259Rev	GTGCGTCGTGGTAGATCTGCT
MT0997For	ACGTCTCGCTGAAGGTGGTC
MT0997Rev	TGCACCCCGGTCACTTGG

### Confirmation of Gene Expression by RT-qPCR

Some genes, which were differentially regulated by exposure to CSC according to RNA-seq data, were selected for confirmation of gene expression levels by RT-qPCR. RNA (500 ng) was reverse transcribed using random primers and the High-Capacity cDNA Reverse Transcription Kit (Thermo Fisher Scientific) according to the manufacturer’s instructions in a Veriti 96-well thermal cycler (Thermo Fisher Scientific). No reverse transcriptase controls were done to confirm the absence of DNA in the RNA samples. PCR was done using 0.5-μM primers (Table 2) and the Applied Biosystems PowerUp SYBR Green Master Mix kit (Thermo Fisher Scientific) according to the manufacturer’s instructions. cDNA was diluted 100-fold before use in the PCR reactions. PCR conditions used were 50°C for 2 min, 95°C for 2 min, followed by 40 cycles of 95°C for 15 s, 60°C for 18 s, and 72°C for 30s. Melt curve analysis was done (95°C for 15 s, 60°C for 1 min, and 95°C for 15 s) to confirm the presence of only one PCR product and the absence of primer dimers. The ΔΔCt method was used and normalization done with *sigA* as a housekeeping gene.

**TABLE 2:** Genes differentially expressed in *Mtb* after 3 and 24 h of CSC exposure.

					**Regulation at 3 h**		**Regulation at 24 h**	
**Gene ID CDC1551**	**Gene ID H37Rv**	**Gene name**	**Function**	**References**	**log2Fold Change**	***p*-value**	**log2Fold Change**	***p*-value**

**Upregulated genes:**								
MT0083	Rv0077c	*ethA2*	Putative oxidoreductase protein	Wohlkonig et al., 2017	4.23	9.31E-45		
MT0148	Rv0140		*Mtb* reactivation-associated proteins	Wayne and Hayes, 1996	2.43	1.12E-02		
MT0196	MT0196	*MymT*	Copper-binding metallothionein	Gold et al., 2008	2.38	4.49E-30		
MT0365^*^	Rv0350	*dnaK*	Important for stress-responsive regulator heat shock protein R (HspR) function	Bandyopadhyay et al., 2012	1.13	5.80E-06		
MT0366^*^	Rv0351	*grpE*	Heat shock stress-responsive element	Bandyopadhyay et al., 2012	1.12	1.08E-05		
MT0397^*^	Rv0384c	*clpB*	Essential stress regulator of *Mtb* and endows survival advantage to dormant bacilli	Tripathi et al., 2020	1.33	1.90E-07		
MT0586	Rv0560c		Methyltransferase	Chen et al., 2019	4.52	2.77E-202	3.53	1.96E-29
MT0706.1	Rv0678		MarR family of transcriptional regulator	Radhakrishnan et al., 2014	–	–	1.33	2.01E-03
MT0838	Rv0816c	*thiX*	Putative thioredoxin	Rahlwes et al., 2020	1.54	1.54E-05		
MT0869	Rv0846c		Putative multicopper oxidase	Rowland and Niederweis, 2012	1.84	4.40E-47		
MT0870	Rv0847	*lpqS*	Probable lipoproteins	Sutcliffe and Harrington, 2004	1.49	5.55E-29		
MT0873	Rv0850		Putative transposase, copper-responsive	Festa et al., 2011	1.05	2.04E-05		
MT0995	Rv0967	*csoR*	Copper-dependent regulation of the copper responsive operon	Liu et al., 2007	2.39	2.63E-03		
MT0996	Rv0968		Hypothetical protein, part of the copper responsive operon	Festa et al., 2011	2.32	1.38E-03		
MT0997	Rv0969	*ctpV*	Putative copper exporter, required for full virulence in *Mtb*	Ward et al., 2010	2.31	9.52E-04		
MT0998	Rv0970		Part of cos operon, responsive to copper	Liu et al., 2007	2.44	5.30E-04		
MT1020^*^	Rv0991c		Redox-regulated molecular chaperone	Becker et al., 2020	1.65	2.40E-08		
MT1068	Rv1039c	*PPE15*	Duplicated from ESAT-6 (esx) gene cluster region 5	Gey van Pittius et al., 2006	1.71	1.21E-03		
MT1259	Rv1221	*SigE*	ECF subfamily sigma subunit E	Manganelli et al., 2001	1.81	3.99E-03		
MT1376.1^*^	Rv1335	*CysO*	Part of the cysteine biosynthesis pathway	Agren et al., 2008	1.13	1.17E-04		
MT1517^*^	Rv1471	*trxB*	Thioredoxin reductase, disulfide reductase	Akif et al., 2008	2.12	1.09E-03		
MT1579^*^	Rv1528c		conserved hypothetical		2.17	3.64E-09		
MT1608	Rv1557	*mmpL6*	Conserved large membrane protein involved in oxidative stress response	Arumugam et al., 2019	2.41	2.04E-35	3.16	7.54E-33
MT1711	Rv1673c		Conserved hypothetical		1.01	6.73E-03		
MT1850	Rv1801	*PPE29*	PPE protein-associated with ESAT-6 (esx) gene cluster region 5	Gey van Pittius et al., 2006	1.52	6.54E-06		
MT1924	Rv1875		Possible pyridoxine 5-phosphate oxidase	Mashalidis et al., 2011	1.23	1.27E-06		
MT2066	Rv2011c		Putative MarR family transcriptional regulator, unknown function	Lee et al., 2016	2.63	3.92E-10		
MT2067	Rv2012		Conserved hypothetical protein		2.38	1.25E-05		
MT2468^*^	Rv2397c	*cysA*	ABC import systems functioning as a sulfate importer	Soni et al., 2020	1.01	4.03E-05		
MT2469^*^	Rv2398c	*cysW*	Sulfur metabolism	Zeng et al., 2013	1.03	2.05E-05		
MT2719	Rv2641	*cadI*	Cadmium-inducible protein	Hotter et al., 2001	2.44	9.77E-02		
MT2780	Rv2707		Cell-binding protein, important in cell invasion	Chapeton-Montes et al., 2008	1.08	2.41E-08		
MT2783^*^	Rv2710	*sigB*	ECF subfamily sigma subunit B	Lee et al., 2008	1.01	2.85E-05		
MT2881	Rv2167c		Probable transposase, DNA damage induced	Boshoff et al., 2003	3.89	4.24E-02		
MT2981	Rv2913c		Probable D-amino acid hydrolase	Av-Gay and Everett, 2000	1.20	3.64E-09		
MT3037	MT3038		Hypothetical protein		1.60	7.51E-11		
MT3038	Rv2962c		Probable glycosyltransferase	Perez et al., 2004	1.82	5.28E-03		
MT3039	Rv2963		Probable integral membrane protein/permeases of unknown function, copper induced protein	Ward et al., 2010	2.43	8.89E-05		
MT3041	Rv2964	*purU*	Formyltetrahydrofolate deformylase	Phong et al., 2015	2.11	8.24E-03		
MT3041.1	MT3041.1		Hypothetical protein		1.86	1.10E-04		
MT3139.1	MT3139.1		Hypothetical protein		1.04	2.51E-16		
MT3140^*^	Rv3054c		Probable NAD(P)H dehydrogenases	Murphy and Brown, 2007	4.68	2.15E-06	1.61	6.20E-05
MT3141	Rv3055		Possible TetR-family transcriptional regulatory protein	Tyagi et al., 2015	1.89	3.46E-15		
MT3142	Rv3056	*dinP*	Y-family DNA polymerases induced upon DNA damage	Kana et al., 2010	1.65	1.32E-15		
MT3150.1	Rv3065	*emrE*	Possible multidrug efflux pump	Poole and Fruci, 2016	1.55	1.58E-13		
MT3151	Rv3066		TetR-like DNA-binding regulator	Bolla et al., 2012	1.33	2.07E-07		
MT3248	MT3248		PPE protein	Karboul et al., 2008	1.38	6.60E-15		
MT3249	Rv3160c		Possible TetR/AcrR transcriptional regulator		2.10	9.87E-30	1.42	6.91E-04
MT3250	Rv3161c		Predicted to encode a dioxygenase, involved in drug resistance	Melief et al., 2019	2.51	1.65E-33	1.69	1.21E-06
MT3263	Rv3174		Putative dehydrogenase/reductase	Bacon et al., 2004	1.80	4.70E-03		
MT3264	Rv3175		Possible amidase (aminohydrolase)		1.44	5.28E-03		
MT3266	Rv3177		Putative peroxidase	Reyes et al., 2012	2.78	4.78E-08		
MT3301^*^	Rv3206c	*moeZ*	Molybdenum cofactor biosynthesis and cysteine biosynthesis	Voss et al., 2011	1.16	2.66E-06		
MT3318	MT3318		Conserved hypothetical		1.25	2.00E-10		
MT3319^*^	Rv3222c		Hypothetical protein sigma factor associated	Thakur et al., 2007	1.03	2.36E-09		
MT3320^*^	Rv3223c	*sigH*	ECF subfamily sigma subunit H	Mehra and Kaushal, 2009	1.04	3.72E-09		
MT3438	Rv3334		MerR transcriptional regulator, linked to early and enduring hypoxic response	Sun et al., 2018	1.47	8.61E-05		
MT3569^*^	Rv3463		Predicted oxidoreductases	Coulson et al., 2017	2.58	3.71E-04		
MT3949	Rv3841	*bfrB*	Ferritin	Sharma et al., 2016	–	–	1.14	0.001109
MT4032^*^	Rv3913	*trxB2*	Thioredoxin reductase	Olson et al., 2013	1.24	3.79E-08		
MT4033^*^	Rv3914	*trxC*	Thioredoxin reductase, disulfide reductase	Akif et al., 2008	1.21	1.68E-06		
**Downregulated genes:**								
MT0356	Rv0341	*iniB*	Glycine-rich cell wall protein which promotes *Mtb* growth in hostile environments (*in vitro*)	Abdalla et al., 2020	−1.39	1.02E-23	−1.67	9.52E-09
MT3830	Rv3727		Similar to phytoene dehydrogenase precursor	Johnston et al., 2010	–	–	−1.83	1.79E-10
MT3831	Rv3728		Possible sugar transporter, major facilitator superfamily	De Rossi et al., 2002	–	–	−1.83	1.38E-12

*Indicate genes directly or indirectly regulated by SigH.

### Statistical Calculations

All graphs were plotted using GraphPad Prism 8.02 or iDEP91 software available online, as indicated above (Ge et al., 2018). Multiple *t*-tests were done, without assuming equal deviation or correction for multiple comparisons, and *p* ≤ 0.05 was considered statistically significant.

## Results

### Effect of Cigarette Smoke Condensate on *Mycobacterium tuberculosis* Growth and Survival

**FIGURE 1:**
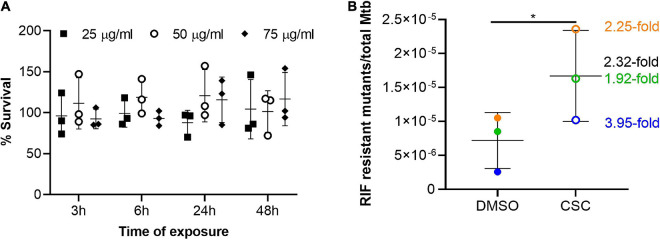
Survival and mutation frequency in *Mtb* exposed to CSC. **(A)** Survival of *Mtb* upon exposure to 25, 50, and 75 μg/ml CSC for 3, 6, 24, and 48 h relative to survival upon exposure to the diluent, DMSO, which was set as 100%. Multiple *t*-tests were done (*n* = 3), without assuming standard deviation and without correction for multiple comparisons. No significant differences were observed. **(B)** Frequency of RIF-resistant mutants, determined by CFU on 7H11 agar RIF (2 μg/ml), compared to bacterial count in the culture, determined by plating dilution series on 7H11 agar. Three independent experiments (*n* = 3) were done and are indicated in different colors. The fold difference in mutation frequency between CSC and DMSO is indicated in the corresponding color with black indicating the mean fold change. The error bars indicate the mean and standard deviation. A paired, two-tailed *t*-test indicated significant increase in mutation frequency upon exposure to CSC (*p* = 0.034). *p < 0.05.

The effect of nicotine, one of the cigarette smoke components, on non-tuberculous mycobacteria and CSC on *Mtb* was previously investigated and shown to have no effect on the growth *in vitro* (Greenstein et al., 2011; Cholo et al., 2020). Since the effect of CSC was previously investigated in H37Rv strain and in 7H9 OADC media (containing catalase which mops up reactive oxygen species), we determined the effect of CSC on the growth and survival of the CDC1551 strain in the absence of catalase to determine the concentrations of CSC to be used in this study. Firstly, we investigated the concentration at which CSC inhibits *Mtb’s* growth by doing MIC assays. The growth of *Mtb* was present in all wells containing only 7H9 dextrose. No growth was observed at a CSC concentration of 125 μg/ml, while growth was still present at 62.5 μg/ml, making the MIC between 62.5 and 125 μg/ml. This was as a result of CSC and not the diluent, DMSO, since growth was observed up to 3.125% DMSO and only 0.78% DMSO was present in the 62.5-μg/ml CSC well. Based on the MIC, *Mtb* was exposed to 25, 50, and 75 μg/ml CSC or DMSO (0 μg/ml) for 3, 6, 24, and 48 h in 7H9 dextrose media. Survival was assessed by CFU enumeration and relative to DMSO exposure, which was set as 100% survival. No significant difference in bacterial survival upon exposure to CSC was observed at the different CSC concentrations or time points tested (Figure 1A).

### Effect of Cigarette Smoke Condensate on Mutation Frequency of *Mycobacterium tuberculosis*

Since CSC contains a whole array of toxic compounds (Borgerding and Klus, 2005; Kim et al., 2018) and was shown to be mutagenic in other bacteria (Claxton et al., 1989; Aufderheide and Gressmann, 2007, 2008; Miyahara et al., 2011), the effect of *Mtb* on mutation frequency was investigated. Growth on RIF was considered as genotypic development of RIF resistance. The frequency of RIF mutations in the presence of CSC (50 μg/ml) was compared to the frequency of RIF mutations in the diluent of CSC, DMSO. The mutation frequency in the presence of CSC was significantly (2.32-fold) higher than in DMSO (Figure 1B).

### Transcriptional Response to Cigarette Smoke Condensate

**FIGURE 2:**
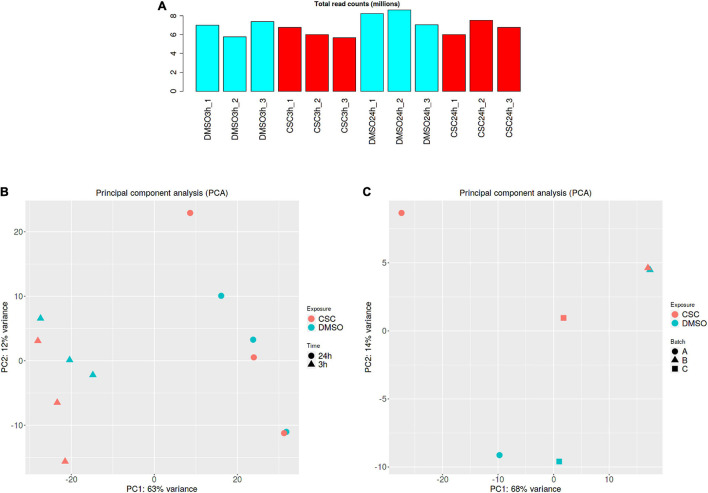
RNA-seq analysis. **(A)** Total read count per million for all samples. **(B)** PCA separated by CSC exposure followed by time for all the samples **(C)** PCA for 24-h samples indicating divergence of the batch B samples (batch A = replicate 1, batch B = replicate 2, and batch C = replicate 3). DMSO samples are indicated in blue and CSC in red. Graphs were generated using the iDEP91 software.

RNA was extracted from *Mtb* cultures exposed to 50 μg/ml CSC and DMSO for 3 and 24 h. To investigate the transcriptional response of *Mtb* to CSC, RNA-sequencing was done and reads mapped to the CDC1551 reference genome. Comparable total reads per million counts were observed for all the samples (Figure 2A). PCA showed that time was the biggest factor in differential gene expression (Figure 2B). This is likely due to multiple factors including the composition and concentration of CSC that likely changed as a result of the volatile nature of CSC. Bacterial growth was also observed previously between the 3- and 24-h time points, contributing to the different bacterial state and transcription at the two time points. Larger variations may also mask some smaller differences in the effect of CSC during the analysis. A separate analysis of the 3- and 24-h data was therefore done. Of the 4,007 genes for which expression was assessed at 3 h of exposure, 3,968 genes passed CPM filtering. Clustering of CSC-exposed and DMSO-exposed replicates/batches was observed. For the 24-h-exposed samples, PCA indicated clustering for replicates 1 and 3 (batches A and C) while replicate 2 (batch B) did not cluster as expected (Figure 2C). Replicate 2 samples were therefore excluded from all subsequent analyses for the 24-h samples. Of the 4,007 genes identified in the remaining four 24-h samples, 3,939 genes passed the CPM filter.

Following exposure to CSC for 3 h, 59 genes were upregulated more than twofold while only one was downregulated (Table 2). Genes upregulated upon exposure to CSC at 3 h included those encoding sigma factors [MT3320 (*sigH*), MT1259 (*sigE*), MT2783 (*sigB*), and MT3319], ESAT-6-associated proline–proline–glutamate (PPE) proteins [MT1068 (PPE15) and MT1850 (PPE29)], and other virulence and reactivation-associated genes [MT1608 (*mmpl6*) and (MT0148)]. Multiple genes which are regulated by SigH were upregulated, including genes encoding oxidoreductases (MT3569 and MT0083) and thioredoxin reductases [MT0838 (*thiX*), MT1517 (*trxB*), MT4032 (*trxB2*), and MT4033 (*trxC*)]. Genes encoding proteins involved in stress response such as MT0365 (*dnaK*), MT0366 (*grpE*), MT0397 (*clpB*), and MT3438) and genes previously shown to be induced by DNA damage [MT2881 and MT3142 (*dinP*)] were upregulated.

**FIGURE 3:**
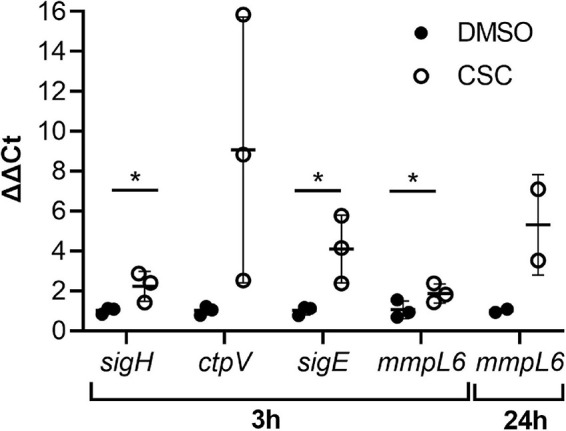
Expression levels of selected genes confirmed by RT-qPCR. The ΔΔCt values are indicated (*n* = 3) and were calculated relative to the housekeeping gene, *sigA*. *SigA* was shown to be consistently expressed in the RNA-seq analysis. Multiple unpaired, one-tailed *t*-tests were done, and *p* ≤ 0.05 was considered statistically significant (*sigH* at 3 h: DMSO vs. CSC, *p* = 0.049; *ctpV* at 3 h: DMSO vs. CSC, *p* = 0.086; *sigE* at 3 h: DMSO vs. CSC, 0.043; *mmpL6*, 3 h, DMSO vs. CSC, 0.048, 24 h, *p* = 0.125). *p < 0.05.

Since heavy metals are a known component of cigarette smoke (Borgerding and Klus, 2005), it was not surprising that the copper-responsive regulon and other genes encoding copper responsive/export systems [MT0995 (*csoR*), MT0996, MT0997 (*ctpV*), MT0998, MT0196 (*mymT*), MT0869, MT0873, and MT3039] as well as molybdenum [MT3301 (*moeZ*)] and cadmium [MT2719 (*cadI*)] responsive genes were upregulated by CSC. At 24 h of exposure to CSC, only seven genes were upregulated and three downregulated. Only five genes, which were upregulated at 3 h of CSC exposure, remained upregulated at 24 h including a methyltransferase (MT0586), MT1608 (*mmpL6*), a probable dehydrogenase MT3140, possible dioxygenase (MT3250), and TetR/MarR regulator (MT3249) encoding genes. Two genes encoding MarR family transcriptional regulators and bacterioferritin [MT0706_1 and MT3949 (*brfB*)] were upregulated at 24 h, but not 3 h of CSC exposure. RNA-seq results were confirmed by RT-qPCR for selected genes (Figure 3).

## Discussion

The significant link between cigarette smoking and TB disease has seen research into the effect of cigarette smoke on host factors. Although an effect of cigarette smoke on the causative agent, *Mtb*, has been suggested (McGrath et al., 2014) and this effect has been investigated on other respiratory bacteria, no studies have, to our knowledge, investigated the effect of CSC on the transcriptional profile or mutagenesis of *Mtb*.

Cigarette smoke condensate inhibited *Mtb’s* growth at a concentration between 62.5 and 125 μg/ml in *in vitro* cultures. *Mtb*’s survival upon exposure to CSC was not significantly affected, although slightly lower CFUs were observed upon CSC exposure compared to DMSO (Figure 1A). CSC previously did not affect the growth of *Mtb* H37Rv in planktonic 7H9 OADC cultures in 96-well plates (Cholo et al., 2020). One of the main components of cigarette smoke, nicotine, did not affect growth of non-tuberculous mycobacteria in culture (Greenstein et al., 2011) but did promote the growth of *Mtb* in epithelial cells (Miramontes et al., 2021). An initial bacteriostatic effect of CSC, within the first 24 h, was observed during survival assays, although doubling of bacteria occurred after 48 h. This is likely due to the dissipation, degradation, and/or chemical changes of the volatile compounds within CSC within the 24-h period. Additionally, interaction of CSE with plastic containers and degradation and/or evaporation of CSE was previously shown to influence the bioavailability of the cigarette smoke components in cell culture experiments (Bourgeois et al., 2016). The volatile nature of CSC is therefore a limiting factor when attempting to explore the effects of more long-term CSC exposure on *Mtb*. It would be interesting to investigate the effects of cigarette smoking on *Mtb* load and virulence factors in animal models such as a macaque model, which closely mimics the immune response in humans.

A 2.32-fold higher mutation frequency was observed after 24 h of exposure to CSC compared to exposure to DMSO (Figure 1B). Strain background, CSC concentration, composition, and time of exposure likely influenced the mutability of *Mtb* upon exposure to cigarette smoke (Mizusaki et al., 1977; Morin et al., 1987; Camoirano et al., 1994; Yim and Hee, 2001; Yim et al., 2002; Aufderheide and Gressmann, 2007, 2008; Bourgeois et al., 2016; Roemer et al., 2016; Thorne et al., 2016). It was also shown that the effect of CSE on bacteria was highly dependent on the bacterial-load-to-CSE ratio (Bourgeois et al., 2016). The effect of the ratio of *Mtb* to CSC concentration, bacterial growth phase, and strain background on the mutability of CSC should therefore be investigated in future studies.

Initially, the intention was to investigate the mutation rate and not just frequency. Preliminary experiments, however, indicated a negative mutation rate (data not shown), likely as a result of bacterial growth and death rate, since these factors had a significant effect on mutation rate calculations in previous studies (Frenoy and Bonhoeffer, 2018). A slight drop in the *Mtb* CFU was observed upon exposure to CSC, although this death did not cause significant percentage survival differences compared to DMSO controls (Figure 1A). Traditionally Luria–Delbrück assays, which use extremely low bacterial counts (thereby allowing exclusion of preexisting mutants from the cultures) and culturing for a prolonged time, are done to assess the mutation frequency and rate. This technique was, however, not usable due to the extremely volatile nature of CSC and inability to culture a low bacterial burden culture for an extended time. Another study has since also used cultures at mid-log growth for mutation rate analysis (Naz et al., 2021). Due to the volatile nature of CSC, the compounds to which *Mtb* were exposed likely also changed during the exposure time, thereby making the exposure conditions variable and mutation rate assessment challenging, if not impossible. Due to its slow growth, the mutation rate of *Mtb* CDC1551 is extremely low.

Mutation rate is also dependent on the drug used for mutation assessment (Ford et al., 2013). The use of phenotypic RIF resistance as a sole indicator of mutations in *Mtb* and accepting genotypic drug resistance without confirming *rpoB* mutations in the *Mtb* colonies (which grew on the RIF plates) are therefore major limitations of this study. The increased mutation frequency determined here does, however, suggest that cigarette smoke could have mutagenic effects on *Mtb*, which could lead to drug resistance. This is in agreement with what was previously observed for other bacteria (Claxton et al., 1989; Aufderheide and Gressmann, 2007, 2008; Miyahara et al., 2011). It is therefore necessary to assess mutation frequency by whole-genome sequencing, where mutations in multiple genes are detected. Mutations driven by CSC which could lead to *Mtb* drug resistance may not be related to known mutations in drug resistance genes, but instead to changes in the cell wall or other virulence factors of *Mtb*. Using whole-genome sequencing will provide the most complete understanding of the mutagenic properties of CSC on *Mtb*. Since the cell wall of *Mtb* is unique (Alderwick et al., 2007) and cigarette smoke has been shown to change the charge on other bacterial surfaces (Crotty Alexander et al., 2015; Hwang J. H. et al., 2016), allowing evasion of the host immune system, it would also be interesting to explore the effect of CSC on the *Mtb* cell wall.

It is also important to remember that CSC contains only the insoluble fractions of cigarette smoke and although it contains many of the chemicals that make up cigarette smoke, limited water-soluble chemicals would be present due to the preparation method (Borgerding and Klus, 2005; Stampfli and Anderson, 2009; Kim et al., 2018). CSC was chosen since it is commercially available and we made use of a single batch during experiments to ensure consistency in its composition. Other methods would require smoking of cigarettes and collection of the water-soluble fractions (CSE), but this would limit the consistency of the composition of the preparation between laboratories and batches and was therefore avoided.

The transcriptional response of *Mtb* to CSC was explored. During the 3-h exposure, the expression of numerous genes was upregulated, while only one was downregulated (Table 2). This likely represents the immediate response to the toxic compounds and oxidative and nitrosative stress generated by CSC. By 24 h, the expression of only five of the 59 genes upregulated at 3 h remained upregulated. The transcriptional response to CSC therefore seems to be a more immediate and short-lived response.

Interestingly, *sigH*, a global regulator (Manganelli et al., 2002), and its downstream regulon were induced by CSC (Table 2). *SigH* is a global stress response regulator which is induced upon exposure to oxidative and nitrosative stress (Mehra and Kaushal, 2009). Additionally, *mmpL6*, encoding for conserved large membrane proteins, which is also involved in oxidative stress response (Arumugam et al., 2019), was upregulated at both 3 h and remained upregulated at 24 h of CSC exposure (Table 2). Increased oxidative stress was also previously suggested to occur due to exposure to the same CSC (the same preparation used in our study) in *Mtb* H37Rv, since catalase, known to remove oxidative stress, caused significant attenuation of the CSC-mediated changes in biofilm formation in *Mtb* (Cholo et al., 2020). The upregulation of oxidative stress response genes is also consistent with the increased oxidative stress observed in other bacteria upon cigarette smoke exposure (Baboni et al., 2010; Goldstein-Daruech et al., 2011; Kulkarni et al., 2012; Hwang J. H. et al., 2016; Chien et al., 2020).

Upregulated genes also included genes known to be directly transcribed under the influence of SigH, e.g., *trxB2*/*trxC* operon which encodes for thioredoxin/thioredoxin reductases that play a role in mitigating oxidative stress and downstream sigma factors *sigE* and *sigB* which further amplify the antioxidative function of *sigH*. Also induced was the expression of heat shock/chaperonin proteins *dnaK* and *grpE*, and *clpB*, which are critical in resolving protein aggregates that may result during oxidative stress. These genes are coregulated with SigH and SigE (Mehra and Kaushal, 2009). The SigH and SigE regulons are two of the most important regulons allowing response to oxidative stress in *Mtb* (Voskuil et al., 2011). Sixteen genes that are known to be controlled by SigH were upregulated by exposure of *Mtb* to CSC indicating true upregulation of a regulon.

Interestingly, RNA-seq results did not show changes in expression of genes involved in the hypoxia response, e.g., *dosR*, *dosS*, or *dosT* (Mehra et al., 2015). Since SigH is required for pathology in the murine model (Kaushal et al., 2002), and for the complete virulence (bacterial burden as well as pathology) in the non-human primate model (Mehra et al., 2012), clearly the antioxidative function promoted by SigH provides *Mtb* with a clear survival advantage during the innate phase of the infection (Kernodle, 2012). Several other studies point to the importance of SigH and its antioxidant function in the virulence of *Mtb*. Thus, it has been established that *in vivo* infection in lungs of mice with *Mtb* leads to accumulation of frameshift mutations that result in a high SigH expression phenotype, and this correlates with greater virulence (Safi et al., 2019). Similarly, it has been suggested that multiplication of genome copies of SigH and the resulting increase in antioxidant activity are responsible for the ablation in the vaccine efficacy of BCG (Sadagopal et al., 2009). Not only is SigH critical for complete TB virulence and lung granuloma formation, but also lack of genes downstream of this key regulator results in disease ablation. Thus, a Δ*sigE* mutant is also attenuated in animal models (Manganelli et al., 2001), while lack of thioredoxins or their cognate reductases results in control of infection and lack of disease (Lin et al., 2016). Similarly, the Δ*sigH* mutant is efficient as a vaccine in protecting both rhesus (Kaushal et al., 2015) and cynomolgus (Singh et al., in process) macaques against lethal challenge with *Mtb*.

Our results therefore indicate that CSC does not cause a hypoxic influence on *Mtb* but rather generates an oxidative stress response. The high-SigH expression phenotype is clearly associated with greater virulence in multiple animal models and has been postulated to correlate with drug tolerance, treatment failure, and relapse of human TB (Safi et al., 2019). While our results were not performed in the context of *Mtb* contained within host phagocytes, they have significant implications. Induction of SigH-mediated response by CSC in *Mtb* suggests that chronic exposure to cigarette smoke may provide a pathogenic advantage to *Mtb* in individuals with pulmonary TB (which may be, in part, driven by increased sigH expression *in vivo*). An investigation of the effect of cigarette smoke on *Mtb* gene expression should therefore be explored in animal models of cigarette smoke exposure. This would allow controlled exposure and assessment of the effect on both host and bacteria for short-term cigarette smoke exposure. It would also be interesting to investigate the oxidative stress response of *Mtb* collected from TB patients who are long-term smokers and compare to *Mtb* from shorter-term smokers or non-smokers.

Cigarette smoke condensate contains numerous heavy metals including cadmium, lead, copper, zinc, and selenium. The average concentrations of these metals are also higher in the blood of smokers than non-smokers (Massadeh et al., 2010). The initial induction of the copper exporter MT0997 (*ctpV*) at 3 h of CSC exposure may have caused loss of numerous metals due to non-specific metal export (Agranoff and Krishna, 2004). Since CSC contains iron and iron drives Fenton chemistry and causes oxidative stress, *brfB* upregulation at 24 h would prevent iron-mediated oxidation, thereby protecting lipids, DNA, and proteins from the potentially toxic effects of iron (Sharma et al., 2016). The expression of copper response regulator CsoR, Rv0970, another cos response member gene, and *cadI*, which responds to cadmium, was also induced in *Mtb* treated with CSC.

This study indicates that the effect of cigarette smoke on the host immune response is not the only factor which is driving the link observed between smoking and TB. The effect of cigarette smoke on the bacteria themselves may very well be a driving force in the increased risk to develop TB, lack of treatment efficacy, and increased mortality observed in smokers. This may in part occur due to cigarette smoke driving genetic mutations as well as inducing virulence factors in the bacteria, thereby allowing more effective bacterial survival.

## Data Availability Statement

The datasets presented in this study can be found in online repositories. The names of the repository/repositories and accession number(s) can be found below: https://www.ncbi.nlm.nih.gov/genbank/, GSE172041.

## Author Contributions

DK contributed funding and overall concept and design, and edited and wrote the manuscript. DW performed all research, conceptualized the study, analyzed the data, and wrote the manuscript. CM validated some data. SM provided resources, funding, and concept and edited and wrote the manuscript. All authors contributed to the article and approved the submitted version.

## Conflict of Interest

The authors declare that the research was conducted in the absence of any commercial or financial relationships that could be construed as a potential conflict of interest.

## Publisher’s Note

All claims expressed in this article are solely those of the authors and do not necessarily represent those of their affiliated organizations, or those of the publisher, the editors and the reviewers. Any product that may be evaluated in this article, or claim that may be made by its manufacturer, is not guaranteed or endorsed by the publisher.

## Footnotes

1
https://mycobrowser.epfl.ch/
